# A randomized comparison of flow characteristics of semiskeletonized and pedicled internal thoracic artery preparations in coronary artery bypass

**DOI:** 10.1186/s13019-017-0589-1

**Published:** 2017-05-16

**Authors:** Opas Satdhabudha, Narupa Noppawinyoowong

**Affiliations:** Department of Surgery, Faculty of Medicine, Thammasat University, 95 Phahonyothin Road, Khlong Nueng, Khlong Luang, Pathum Thani 12120 Thailand

**Keywords:** Semiskeletonization, Diastolic filling, Coronary artery bypass, Internal thoracic artery

## Abstract

**Background:**

Harvesting the internal thoracic artery (ITA) with semiskeletonization is an alternative technique between conventional wide pedicle and skeletonization. It is almost as simple as pedicle harvesting; however, it is supposed to provide the advantage of graft flow and length. Since the heart is unique being the only organ which is perfused during diastole, for comparing the intraoperative graft flow characteristics of semiskeletonization and pedicle technique, we used diastolic filling (DF) using transit-time flow measurement as a primary result. The objective of this study is to compare if semiskeletonized ITA has a greater effect on the intraoperative DF of graft flow versus conventional pedicled ITA in coronary artery bypass.

**Methods:**

Between July 2015 and May 2016, a prospective evaluation of 60 consecutive patients undergoing coronary artery bypass grafting for left anterior descending artery revascularization were randomized to having semiskeletonized (*n* = 30) or conventional pedicled (*n* = 30) ITA graft harvested by the same surgeon. Intraoperative transit-time flows were obtained. The DF of the ITA graft at the end of operation was evaluated in two groups.

**Results:**

The intraoperative DF was significantly greater in the semiskeletonized grafts than in the pedicled grafts (70.50 ± 14.15 versus 57.6 ± 19.39%; *p* = 0.005). No statistical difference was observed comparing quantitative pulsatile flow and pulsatile index at the end of the operation in the two groups. However, the free flow of the conduit during the cardiopulmonary bypass before the anastomosis performed was greater in semiskeletonized group than in pedicled group (94 ± 48.37 versus 56.35 ± 34.90 ml/min; *p* = 0.003). The total operative time was comparable between two groups (*p* = 0.092).

**Conclusions:**

Semiskeletonized ITA resulted in superior DF of left anterior descending bypass graft flow as compared with pedicled ITA. It is also provide a greater free flow and length of the graft without the long-delayed operative time.

**Trial registration:**

Trial registration number (Study ID): TCTR20160913002

Date of registration: September 10, 2016

## Background

There are clear benefit to using the left internal thoracic artery (ITA) as a bypass graft to the left anterior descending artery (LAD) evident by valuable outcomes [1–3]. Generally, there are two well-established ITA harvesting techniques, pedicle and skeletonization, with different effects. Conventional pedicle technique is a simple and feasible technique for daily practice. However, the wide-pedicled graft with surrounding muscle and fascia has limited arterial length, especially in cases where multiple anastomoses are required. Recent studies have reported that skeletonization of the ITA can improve conduit flow with larger vessel diameter, has increased length, and can reduce risk of deep sternal infection in diabetic patients [4–8]. However, the skeletonization technique is not the preferred choice of some surgeons because of potential injury to the artery and the increased time and focus required versus the pedicle technique. Harvesting the ITA with semiskeletonization techniques has been first described by Horii and Suma in 1997, this has the advantages of both the wide pedicle and the full skeletonization technique [9]. Semiskeletonization provides a lean-pedicled graft with maximum length, without the lengthen operative time. However, a study is needed to support the supposition that semiskeletonized graft has an advantageous effect in terms of graft flow augmentation.

The objective of this study was to compare whether semiskeletonization has a greater effect on the intraoperative graft flow than conventional pedicled ITA in coronary artery bypass. Recently, intraoperative transit-time flow (TTF) measurement has been considered a reliable method for assessing internal thoracic artery and coronary artery bypass graft flow. Routine TTF measurement is advocated by several authors. It represents a reproducible method, and provides three parameters: the mean quantitative flow, the pulsatility index (PI) and the diastolic filling percentage (DF) for intraoperative evaluation of graft function [10–12]. The coronary perfusion of the heart is uniquely higher during diastole, which is the opposite of all other vascular beds in the body. In order to compare the intraoperative characteristics of two particular harvesting techniques, this randomized study was then designed to primarily evaluate the difference in DF, which is one of the introductory studies to use this parameter to evaluate the grafts.

## Methods

Between July 2015 and May 2016, a prospective evaluation of 60 consecutive patients undergoing coronary artery bypass grafting for LAD revascularization was randomized to having left ITA harvest semiskeletonized (S group, *n* = 30) or pedicled (P group, *n* = 30) by the same surgeon. Exclusion criteria were (1) emergency surgery, (2) ejection fraction < 0.5, (3) combined cardiac-associated operative procedure, and (4) left ITA diameter < 1.5 mm. Randomization was performed by opening a sealed envelope at the time of incision. The intraoperative primary outcome involved measurement of diastolic filling of ITA graft by a transit-time flowmeter. In addition, graft flow characteristics, length of graft, and operative time also were monitored.

This study has been approved by the Human Ethics Committee of Thammasat University (MTU-EC-SU-1-097/58).

### Operative techniques

A median sternotomy was performed. The left hemisternum was elevated by using an asymmetric sternal retractor. The reflection of the mediastinal pleura was dissected away from the anterior chest wall. The ITA and accompanying veins were identified beneath the endothoracic fascia by direct visualization and manual palpation. The ITA conduit was mobilized along its entire course from the first rib to the sixth rib nearby the epigastric bifurcation.

#### Semiskeletonized harvesting

The opening incision of the endothoracic fascia was started at the third intercostal space longitudinally and medially of the medial accompanying vein with using electrocautery at low energy setting (force 20, coagulate, Valleylab). Downward traction of the fascia, which was still attached to the anterior chest wall, was applied. The ITA pedicle, with accompanying veins, was mobilized from the chest wall, then surrounding muscle and fascia was scraped off with a cold cautery tip. ITA’s branches were proximally ligated with hemostatic clips and distally divided diathermally.

#### Pedicle harvesting

A longitudinal incision was made on the fascia approximately 1 cm medially and laterally along the course of the artery. The conduit was harvested as a pedicle including the fascia, muscle, connective tissue, and accompanying veins. The entire pedicle was detached from the chest wall. ITA’s branches were secured with clips or electrocauterization (force 25, coagulate, Valleylab).

With both harvesting techniques, care must be taken not to grasp the artery directly; retraction against the vein or fascia was mandatory. The branches were ligated with hemostatic clips. After full heparinization for 5 min, the graft was taken down by transection at the distal end, just 5 millimeters proximal to its epigastric bifurcation to preserve the collateral of the superior epigastric and musculophrenic arteries.

### Measurements of ITA graft flow

ITA flow was measured in five separate circumstances (Table 1). F1 to F4 were of measurements before the ITA-LAD grafting was performed. F1 and F2, in situ pulsatile flows, were mean quantitative flow measured by using a flowmeter (the VeriQ system, Medi-Stim from Norway, Model VQ4122) consisting of a measuring probe available in different sizes varying from 1.5, 2.0, 3.0 mm graft diameter in the middle third of the artery. F1 represented the mean native ITA flow at the beginning of harvest. F2 represented the in situ post-harvest flow after the artery was soaked with topical papaverine for 5 min. F3 and F4, free flows at distal end, were direct measurement of free flow bleeding after distal transection into a calibrated cup over 15 s and at operating room positive pressure 20 Pascal. F4 represented the graft flow reserve prior to the anastomosis during the cardiopulmonary bypass. After the ITA-LAD grafting was completed and the patient came off the cardiopulmonary bypass (CPB), F5 was measured together with the diastolic filling (DF) and the pulsatile index (PI).

**Table 1:** Flow measurements timeline

No.	Time point	Flow type	Measurement	Site on ITA
F1	Beginning of harvest (native ITA flow)	Pulsatile flow^a^	TTF	Middle third
F2	End of harvest	Pulsatile flow^a^	TTF	Middle third
F3	End of harvest	Pulsatile flow^a^	Free flow	Distal end
F4	Before grafting	Non-pulsatile flow^b^	Free flow	Distal end
F5	End of procedure (post-CPB)	Pulsatile flow^a^	TTF	Middle third

^a^Pulsatile flow is patient pulsatile flow, measured at systolic blood pressure of 110–130 mmHg and diastolic blood pressure of 60–80 mmHg

^b^Non-pulsatile flow is pump non-pulsatile flow, measured during cardiopulmonary bypass at maintained mean arterial pressure of 70 mmHg and pump flow of 3.0-3.5 L/min/m2

### Measurements of ITA bed and graft length

**Fig. 1:**
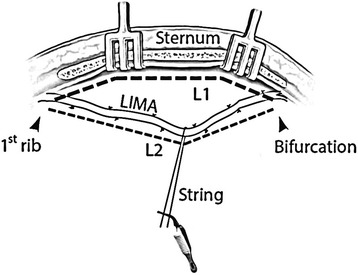
Measurement of the ITA bed length (L1) and graft length (L2). L1 = Sternal length of ITA bed, L2 = ITA graft length from the first rib to the bifurcation

After the ITA was completely dissected from the chest wall, L1 was measured using a silk suture following along the course of ITA bed, from the first rib down to the sixth rib near the ITA bifurcation. L2, the graft length, was measured prior to transection at distal end with a separate silk suture. During the retractor was in place, the left lung and thymic tissue were pushed down to allow the ITA to sag with gravity of bulldog clamp and string. A silk suture was used, following along the artery, to measure its length (Fig. 1). Both strings, L1 and L2, were measured with the same ruler.

### Statistics

Normal distributed continuous data were expressed as the mean ± 1 SD, and Student’s *t*-test was used to compare the two groups. Non-normal distributed continuous data were expressed as a median within the 10^th^ and 90^th^ percentiles range, and the Mann-Whitney test was used to compared the two groups. Nominal data were expressed as frequency and percentage, and the two-tailed Fisher’s test was used for analyses. A value of p less than 0.05 was considered to be statistically significant. IBM SPSS Version 23 was used.

## Results

The patient demographic data demonstrated no statistical differences among the two groups (Table 2).

**Table 2:** Demographic data

Patient characteristic	P group (*N* = 30)	S group (*N* = 30)	*p*-values
Age (years)	64.87 ± 6.94	62.80 ± 9.42	0.337
M:F	16 (53.3%)	18 (60%)	0.794
Weight (kg)	66.39 ± 11.82	68.95 ± 11.70	0.403
Height (cm)	159.40 ± 7.92	162.77 ± 8.32	0.114
Body surface area (m^2^)	1.69 ± 0.17	1.76 ± 0.18	0.108
Smoker	2 (7%)	6 (20%)	0.254
Diabetes	18 (60%)	15 (50%)	0.604
Hypertension	26 (87%)	26 (87%)	1.000
Hyperlipidemia	25 (83%)	20 (33%)	0.233
Family history of CAD	1 (3%)	0 (0%)	1.000
PVD	2 (7%)	6 (20%)	0.254

*CAD* coronary artery disease, *PVD* peripheral vascular disease

### Graft flow measurement

Before harvesting, baseline mean native flow (F1) was 11.69 ± 5.35 ml/min and comparative in both groups (*p* = 0.416). After complete harvesting, the mean flow of the in situ graft (F2) and free flow after transection at the distal vessel end (F3) showed no differences as well. Although there was no statistical difference (*p* = 0.060), our data showed that the free flow (F3) in the semiskeletonized group had the propensity to be greater than in the pedicled group. Before the anastomosis was constructed, the free flow of the conduit during the cardiopulmonary bypass (F4) was nearly 90% greater in the semiskeletonized group than in the pedicled group (96(36-240) versus 51(10-120) ml/min; *p* = 0.004) (Tables 3).

**Table 3:** ITA graft flow measurements

Flow	P group (*N* = 30)	S group (*N* = 30)	*p-*values
Before grafting performed			
F1	11.11 ± 5.17	12.31 ± 5.58	0.416
F2	6.69 ± 2.97	6.18 ± 2.73	0.512
F3	19.15 ± 12.42	27.53 ± 20.38	0.060
F4	56.35 ± 34.90	94 ± 48.37	0.003
After grafting performed; post-CPB			
F5	22.37 ± 19.58	31.60 ± 19.56	0.073
PI	3.05 (1.3–12.8)	2.1 (1.7–4.4)	0.228
DF	57.6 ± 19.39	70.50 ± 14.15	0.005

After the ITA-LAD grafting was completed, and patient came off the cardiopulmonary bypass, the DF was significantly greater in the semiskeletonized group than in the pedicled group (70.50 ± 14.15 versus 57.6 ± 19.39%; *p* = 0.005). No statistical difference was observed comparing mean flow-F5 (*p* = 0.073) and PI (*p* = 0.228) among two groups.

### Graft length and operative time

The length of ITA bed on the chest wall (L1) varied from 12.6 to 17.5 cm (15.15 ± 1.12 cm) with no notable difference between two groups (*p* = 0.623). The length of ITA graft from the first rib to the epigastric bifurcation (L2) was greater in the semiskeletonized group than in the pedicled group (16.06 ± 1.43 versus 14.63 ± 1.29 min, *p* < 0.001). The semiskeletonized ITA length averaged 1.5 cm (10%) longer than the pedicled graft (Table 4).

**Table 4:** ITA length and operative time

	P group (*N* = 30)	S group (*N* = 30)	*p*-value
L1-length of ITA bed	15.22 ± 1.19	15.08 ± 1.05	0.623
L2-length of ITA graft	14.63 ± 1.29	16.06 ± 1.43	<0.001
ITA injury	1 (3%)	0 (0%)	1.000
Operative time	217.23 ± 53.25	240.73 ± 52.98	0.092

There was no difference of the operative time among two groups (*p* = 0.092). Injury to the ITA occurred in one patient (3%) in the pedicled group (*p* = 1.000). Postoperative hemorrhage from the ITA graft-related causes was not observed. None of the patients had development of mediastinitis at 30 days after the operation.

## Discussion

The ITA is an elastic artery in which the diameter varies from 1.9 to 2.6 mm in adults [13]. It arises from the subclavian artery near its origin and crosses the first rib cartilage to enter the thorax. It runs deep into the connective tissue of the endothoracic fascia, but is superficial to the transverse thoracic muscles. It is accompanied by the internal thoracic veins. At termination, it splits into the musculophrenic artery and the superior epigastric artery around the sixth intercostal space.

Using the ITA as a bypass graft to the LAD is advantageous in terms of longevity of patency and patient survival. Traditionally, ITA was harvested as a wide pedicle with surrounding muscle and fascia. In 1987, the skeletonization procedure was first described by Keeley, involving the harvest of only the ITA without any surrounding tissue [14]. After a decade, a new method to skeletonize and harvest the ITA using an harmonic blade was presented by Higami et al in 2000 [15]. Although it has a number of advantages, it requires meticulous technical attention and generally consumes more time than the conventional pedicle technique. However, Kieser et al. revealed that harmonic skeletonization was less time-consuming than conventional skeletonization (mean harvest time of 28.4 versus 32.2 min) and when in a highly-experienced hand, the mean duration of harvest was even reduced to 15.4 min [16].

**Fig. 2:**
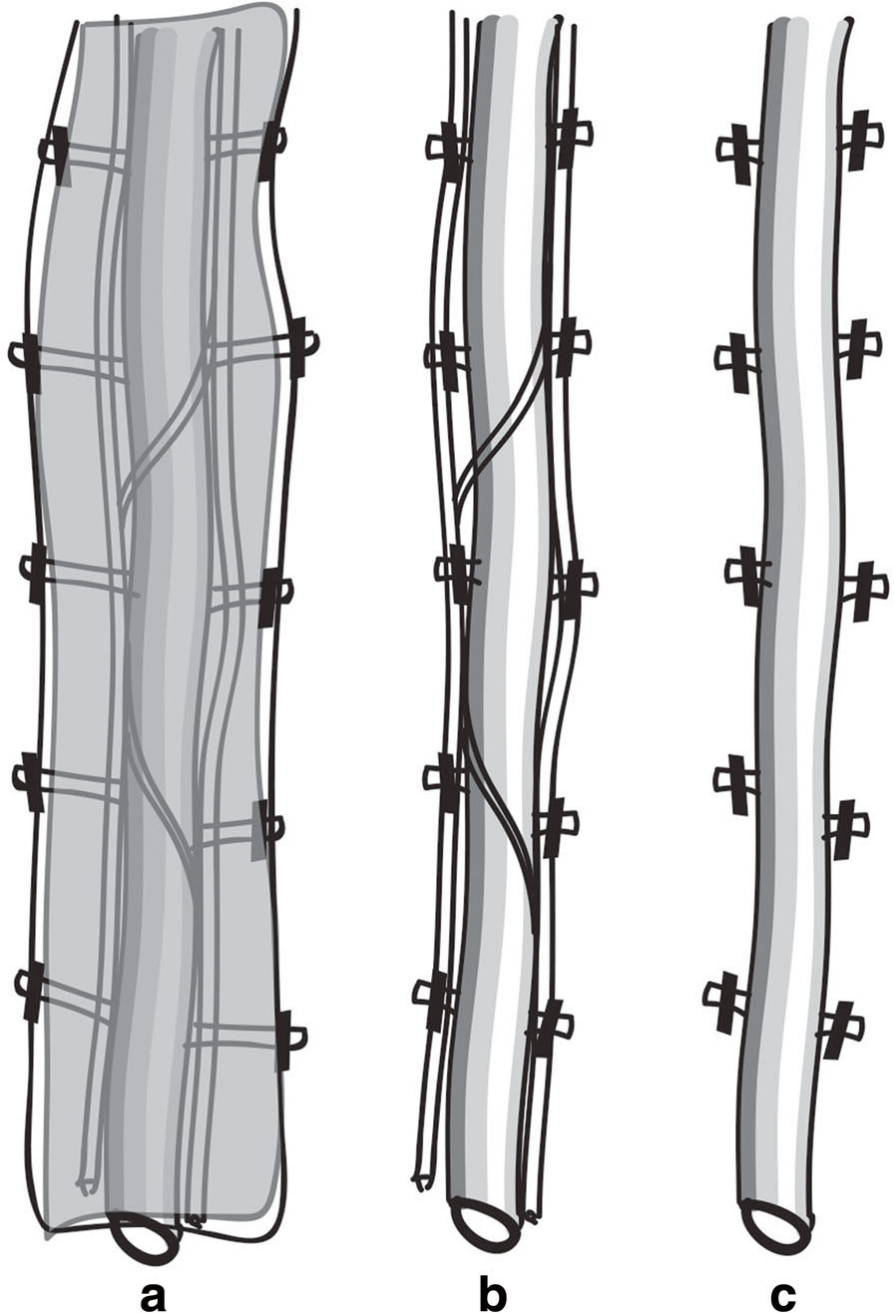
Three different techniques of ITA harvesting. ITA can be harvested in 3 techniques: **a** pedicled, with entire pedicle including veins, muscle and fascia; **b** semiskeletonized, with just two accompanying veins; or (**c**) skeletonized, only the artery is isolated

Harvesting the ITA with semiskeletonization was initially detailed by Horii and Suma in 1997 to gain the advantages of both the conventional pedicle and the full skeletonizing technique [9]. In theory, semiskeletonization is in between pedicle and skeletonization technique (Fig. 2). In semiskeletonization, both the artery and the two accompanying veins are mobilized with no other surrounding tissue, while skeletonization leaves the internal thoracic venous plexus intact [17]. For surgeons familiar with conventional pedicle technique, it is quite similar, but provides some advantages of skeletonization.

At our institute, we have seen that the semiskeletonized graft provides the benefits of a good intraoperative blood flow reserve and added extra length of graft (average 10%). The median free flow rate of semiskeletonized graft (F4) is nearly 90% greater than of pedicled grafts. The total operative time was not noticeably different between both procedures. None of the patients had developed superficial or deep sternal infection in our study.

It was apparent that each time sequences demonstrates characteristic mean flow. The blood flow reduction during the harvesting from F1 to F2 was ascribed to the manipulation-related vasospasm [18]. There was no discernible difference between the two groups. Blood flow reduction of graft at the end of harvesting (F2) was considered independent of manipulation by the semiskeletonization.

In addition, the ITA has an abundant collateral blood supply to its runoff bed, which provides protective effect to its intima [19]. For this reason, ligation of all branches along the artery can be a cause of increasing vascular bed resistance and decreasing graft flow. This is confirmed by an increased flow rate with free bleeding under near zero resistance after transection of the distal vessel end (F3). The free flow bleeding increased by an average of at least three times between F3 and F4; this may be attributed to the reversible effect of vasospasm similarly in both groups.

Compared to the native ITA flow, the graft flow doubly increased after grafting to the LAD (F5). Theoretically, blood flow to the heart occurs mainly during diastole; therefore, diastolic-filling capacity should not be overlooked. The coronary flow pattern is pulsatile through the cardiac cycle, which is accentuated in diastolic phase of relaxation. The flow pattern in a bypass graft should also mainly be diastolic as in a coronary artery. One of the most important causes of reduction of graft flow is native coronary flow competition, which mainly happens in the diastole [20]. The good flow reserve in semiskeletonized group (F4) is then, a favorable effect, especially in the presence of competitive coronary flow. Several factors can have an effect on the graft flow: the quality of native coronary artery, graft flow competition, and possible spasm of the graft or coronary artery. A low flow as such does not necessarily represent quality of the anastomosis [21]. Morota et al. in a swine model study, demonstrated that diastolic filling represents the most reliable indicator of graft patency [22]. The quantification of diastolic filling is supposed to be important in the case of low flow, averaging less than 15 ml/min. Diastolic filling more than 50% means more than half of coronary flow occurred in diastole, which is compatible with the characteristic of coronary artery blood flow.

Other notable data to be considered from TTF is PI. PI is indicated as an important predictive of outcomes. A value of PI between 1 and 5 represent a satisfactory grafting. Mortality following elective CAB was significantly higher in patients with a PI > 5 [11]. In our study, there was slightly higher PI in the pedicled than in the semiskeletonized group; however, it was not of significant difference.

In general, the reactivity of an arterial graft varies along its course. As demonstrated in the ITA, the mid portion of the graft is less reactive than the distal and the proximal portions [23, 24]. Therefore, trimming off the distal end of the graft, together with establishing the shortest possible route to the LAD, may be important and practical for preventing vasospasm of an ITA graft [25]. With semiskeletonization, longer distal portion may be trimmed off because it is easier to measure the precise graft length with the skeletonized ITA. There is no need to leave the graft much longer than expected as does the pedicled graft.

Sternal blood flow significantly decreases after conventional pedicle ITA harvest. Skeletonization can reduce the degree of sternal ischemia and also deep sternal wound complications [8]. In order to preserve the collateral blood flow to the sternum, the ITA branches should be ligated as close as possible during the harvest [26, 27]. However, there has been no study showing reduced deep sternal wound infection following the semiskeletonization.

Harvesting an ITA with semiskeletonization needs 10 to 20 cases to overcome the learning curve. Although it may take more time to harvest an ITA with this technique, the total operative time does not significantly increase. To some extent, when compared with the pedicled technique, semiskeletonization yields a longer ITA graft, making it easier to measure the optimal length before grafting to the LAD. Moreover, semiskeletonization may also yield a larger ITA lumen without consuming as much time preparing the vessel end. This makes anastomosis more straightforward. Last but not least, twisting of the ITA graft rarely occurs.

While it may seem a bit counterintuitive, in semiskeletonization, the ITA can be easily visualized during harvest; therefore, when compared to the pedicle technique, risk of vascular injury is lower. In addition, leaving sufficient perivascular tissue provides the benefit in handling the artery during grafting. Currently, the semiskeletonized technique has become customarily performed in our institute.

## Conclusion

Semiskeletonized ITA results in superior diastolic filling of left anterior descending artery bypass graft flow as compared to the pedicled ITA. It is also effective in providing a greater free flow and length of the graft without the long-delayed operative time. Semiskeletonization appears to offer a beneficial middle ground between the advantages of skeletonization and pedicle technique: sufficient long graft with consistent and feasible practice. However, more studies are needed on the long-term patency of the semiskeletonized technique to determine patient outcomes.

## Abbreviations

CAB: Coronary artery bypass
CAD: Coronary artery disease
DF: Diastolic filling
ITA: Internal thoracic artery
LAD: Left anterior descending coronary artery
PI: Pulsatile index
PVD: Peripheral vascular disease
TTF: Transit-time flow
